# Rapid Learning and Long-Term Memory for Dangerous Humans in Ravens (*Corvus corax*)

**DOI:** 10.3389/fpsyg.2020.581794

**Published:** 2020-10-21

**Authors:** C. R. Blum, W. Tecumseh Fitch, T. Bugnyar

**Affiliations:** ^1^ Department of Cognitive Biology, University of Vienna, Vienna, Austria; ^2^ Haidlhof Research Station, University of Vienna and University of Veterinary Medicine Vienna, Vienna, Austria

**Keywords:** predator recognition, corvid, raven (*Corvus corax*), alarm call, memory, learning, individual human recognition

## Abstract

Like many predatory species, humans have pronounced individual differences in their interactions with potential prey: some humans pose a lethal threat while others may provide valuable resources. Recognizing individual humans would thus allow prey species to maximize potential rewards while ensuring survival. Previous studies on corvids showed they can recognize and remember individual humans. For instance, wild American crows produced alarm calls toward specifically masked humans up to 2.7 years after those humans had caught and ringed them while wearing that mask. However, individual behavior of the crows or the impact of social features on their responses, was hardly examined. Here, we studied predator learning and social effects on responses, using a similar method, in captive common ravens (*Corvus corax*). We investigated learning and the impact of key social components on individual reactions to artificial predators. Human experimenters wore two types of masks while walking past two raven aviaries. In four training trials, the “dangerous” mask was presented while carrying a dead raven, whereas the “neutral” mask was presented empty-handed. Between every training trial and in all following trials, we presented both masks without dead ravens. We assessed the subjects’ (i) learning speed, (ii) selective long-term response, and (iii) potential effects of social dynamics on individual alarm calling frequency. Ravens learned quickly (often based on the first trial), and some individuals distinguished the dangerous from the neutral mask for the next 4 years. Despite having received the same amount and quality of exposure to the dangerous mask, we found pronounced individual differences in alarm calling that were fairly consistent across test trials in socially stable situations: dominance, but not sex explained individual differences in alarm responses, indicating the potential use of alarm calls as “status symbols.” These findings fit to those in wild bird populations and dominant individuals signaling their quality. Changes in the individuals’ participation and intensity of alarm calling coincided with changes in group composition and pair formation, further supporting the role of social context on ravens’ alarm calling.

## Introduction

Learning about new predators allows individuals to adapt existing anti-predator behavior to new threats. Many animal species are able to recognize conspecifics on an individual level (Tibbetts and Dale, 2007; Wiley, 2013), and several taxa have been shown to learn to recognize novel predators on a species level (Griffin et al., 2000). However, studies showing individual recognition of (potentially dangerous) members of other species remain rare. Anti-predator behavior is risky and reduces time and energy for other contexts like foraging and reproduction (Montgomerie and Weatherhead, 1988; see Lima and Dill, 1990 for a review). Limiting predator responses to specific individuals rather than generalizing to an entire species should therefore be adaptive (Berzins et al., 2010). For instance, individuals of the same predator species may differ substantially in their hunting abilities, because of sexual size dimorphism, different levels of experience with prey etc. (e.g., Hakkarainen et al., 1996). Indeed, studies on tits showed them capable of assessing the risk posed by individual predators, for example by adjusting referential warning calls and behavioral responses depending on the predator’s size (Templeton et al., 2005; Courter and Ritchison, 2010). For human individuals, such differences in behavior may even be pronounced: what humans do in interaction with specific individuals of another species can vary substantially, ranging from providing food and shelter to hunting. Several species have adapted to humans’ presence, i.e., urbanization, better than others (Shochat et al., 2006; Kark et al., 2007), and several species in close contact with humans have been shown to recognize human faces (Davis, 2002). Recent studies investigating individual predator recognition, predominantly in birds, therefore used humans as test stimuli (Cornell et al., 2012; Swift and Marzluff, 2015; Lee et al., 2019).

Most birds use mobbing as an anti-predator behavior. Mobbing is a coordinated action of multiple individuals of a weaker species against one or more individuals belonging to a more powerful species (Hartley, 1950). Mobbing behaviors can range from uniform, harsh predator directed alarm calls (scolding) to physical attacks (Altmann, 1956) and primarily serve to harass predators into leaving. Aside of moving off predators, mobbing may also function as signal of (male) quality and/or status (Slagsvold, 1984; Ellis, 2009; Tanager, 2011), and an opportunity for young to learn to recognize predator species (Curio, 1978; Curio et al., 1978a,b). Specifically, corvids have frequently been tested for individual predator learning: American crows (*Corvus brachyrhynchos*) have been shown to learn about novel predators and remember for at least 2.7 years (Marzluff et al., 2010). Experimenters wore masks while catching and ringing wild crows. The directly handled crows remember the masks worn during catching and responded with significantly higher scolding intensity than toward the control masks. Additionally, nearby observer crows who were not handled did so as well. A follow-up study provided experimental evidence of social transmission of predator-knowledge, as individuals not present during the catching event produced alarm calls when confronted with the “dangerous” mask (Cornell et al., 2012). In a second follow-up study, American crows were again exposed to masked humans, this time carrying a dead conspecific (Swift and Marzluff, 2015). The crows responded with alarm calls and avoidance of areas where the presentations occurred, and the response lasted at least 7 weeks. Similar studies on wild jackdaws (*Corvus monedula*) showed that these birds can learn to recognize individual humans by their facial features (Davidson et al., 2015). Experimenters approached jackdaws while wearing two types of masks, one of which was previously worn while handling their eggs; the “dangerous” mask later elicited longer latencies to return to the nest box than the neutral mask. In a further step, playbacks of conspecific alarm calls were coupled with the presentation of a masked human (Lee et al., 2019). In later presentations, without the playback, the birds showed increased latencies to return to their nest boxes when the masked human was nearby, but not when presented with a control mask.

Taken together, these studies provide experimental evidence of predator learning in corvids, specifically when using masked humans as novel predators. Training events like catching or presentation of dead conspecifics (for American crows), handling of the nests or playback of alarm calls (for jackdaws) were restricted to single events or periods lasting no more than 3 days. Yet in all cited studies, obvious differences in behavioral response to the different masks were documented, indicating quick learning capabilities. Because several of these studies have been conducted on wild populations, the control over individual exposure intensity was intrinsically limited (e.g., for crows), or the tests were restricted to short time periods only (e.g., for jackdaws). Hence, individual variation in birds’ anti-predator responses have hardly been investigated for consistency over time and different social settings.

The current study focuses on another member of the corvid family, the common raven (*Corvus corax*). Outside the breeding period, ravens tend to form groups with moderate to high degrees of fission-fusion dynamics. Throughout the day, they split from large roosting-flocks of up to several 100 individuals and forage in sub-groups of varying composition (Braun and Bugnyar, 2012), in which individuals may meet each other repeatedly at one or more locations (Loretto et al., 2017). Depending on the food source and foraging strategies, these sub-groups may range from a few (2–5), to around 20 or up to 100 birds (Marzluff and Heinrich, 1991; Dall and Wright, 2009; Braun and Bugnyar, 2012). It has been hypothesized that these social conditions favor the emergence of sophisticated forms of cognition (Whiten and Byrne, 1988; Dunbar, 1998; but see DeCasien et al., 2017) including long-term memory for individuals (Fiore et al., 2008). Previous studies revealed that ravens possess long-term memory of the relationship valence to former group members (Boeckle and Bugnyar, 2012). Social context and group compositions also affect ravens’ risk-taking behavior (Stöwe et al., 2006). Furthermore, a series of studies indicated that ravens can pay close attention to human facial features like gaze direction (Bugnyar et al., 2004; Schloegl et al., 2007), making them well-suited for the purpose of our study: long-term memory for heterospecific individuals (in this case, humans).

Similar to the work on crows and jackdaws (e.g., Swift and Marzluff, 2015; Lee et al., 2019), we had a human presenter wearing one of two types of masks: one mask was worn with the experimenter carrying an unfamiliar dead raven in one hand, simulating the outcome of a predation event; the other “neutral” mask was worn by an experimenter with both hands empty. Unlike the previous studies, we tested captive birds in their social groups, i.e., the presenter walked past the aviaries of a captive raven colony. We thus had full control over each individual’s exposure to the training stimulus, which allowed us to examine individual variation in the ravens’ responses within and across experimental presentations and to investigate the effects of individual and social features on alarm calling participation. Notably, we tested the ravens’ discrimination between the “dangerous” and neutral mask on a long-term basis, by presenting both masks without reinforcement (i.e., experimenter empty-handed) for 4 years. During this time, group compositions changed from two initial groups of eight individuals each, to one large group of 12, and finally to multiple pairs. In the first 3 years, we also recorded focal protocols analyzing daily life situations, from which we extracted information about dominance relationships.

We predicted that the ravens would quickly learn to discriminate between masks, leading to higher scolding intensities (i.e., longer duration of alarm calling) for the dangerous mask than for the neutral mask. Based on previous reports and own pilot observations, we also predicted substantial individual variation in alarm calling intensity, potentially explained by individual-specific features like sex, raising type, and kinship, and/or by social features like group composition and dominance. Based on previous findings in corvids, we hypothesized that ravens would continue discriminating between the masks over a long time period, possibly years, without reinforcement (i.e., without the pairing with a dead raven). Furthermore, we expected that individual variation in scolding would be consistent across experimental presentations, as long as the group composition remained stable.

## Materials and Methods

### Ethical Note

This experiment was approved by the animal ethics and experimentation board of the University of Vienna under the license number 2018-011. The entire data collection was non-invasive.

### Subjects and Housing

Study subjects were 16 captive ravens (Table 1) housed in two large aviaries at the Haidlhof Research Station, an outdoor facility of the University of Vienna and the University of Veterinary Medicine, Vienna, located near Bad Vöslau, Lower Austria. At the begin of the study in 2011, birds were kept in two social groups of eight subjects each: Group A consisted of five females and three males; they were the offspring of four captive breeding pairs, were raised from hatching to fledging by their parents in 2010 and arrived at Haidlhof in September of that year. Group B consisted of four females and four males; they originated from captive and wild breeding pairs (three and five birds, respectively) and were raised to fledging by their parents (two) or human foster parents (six in total). Two hand-raised females hatched in 2010, all others hatched in 2011 and arrived at Haidlhof in September of that year. Over the years, all ravens were exposed to changes in group composition and size, simulating the dynamics under natural conditions (compare Braun and Bugnyar, 2012) and adhering to the birds’ maturation and their transition from non-breeding to breeding state (compare Heinrich, 1999). In October 2012, four birds of Group A left the station, and the remaining individuals were merged into one group. Over the following 2 years, the non-breeder group consistently became smaller as individuals pair-bonded and were transferred into separate compartments for breeding. Pairs continued to be included in the experiment as long as they were kept at Haidlhof. Five individuals left the station in 2014 and three more in 2015; 2015 represents the end of this study as only two birds remained at Haidlhof the following years.

**Table 1:** List of individuals. For ease of identification, single-individual sib-groups were named after individuals.

Name	Initial group	Sex	Year hatched	Raising	Sib-group
Anton	A	Male	2010	Parent-raised	3
Ellen	A	Female	2010	Parent-raised	4
Heidi	A	Female	2010	Parent-raised	3
Jakob	A	Male	2010	Parent-raised	4
Jonas	A	Male	2010	Parent-raised	2
Klara	A	Female	2010	Parent-raised	4
Lena	A	Female	2010	Parent-raised	1
Sophie	A	Female	2010	Parent-raised	1
Astrid	B	Female	2010	Hand-raised	2
Joey	B	Female	2010	Hand-raised	Joey
Lellan	B	Female	2011	Hand-raised	Lellan
Matte	B	Male	2011	Hand-raised	Matte
Orm	B	Male	2011	Hand-raised	Orm
Ray	B	Male	2011	Hand-raised	Ray
Skadi	B	Female	2011	Parent-raised	5
Thor	B	Male	2011	Parent-raised	5

All birds were marked with colored rings for individual identification. Each aviary had smaller chambers attached that provided opportunity for shelter and visually isolated retreating opportunities, but remained closed during experiments. Multiple branches provided enrichment and perching opportunities. The ground substrate consisted of gravel, wood chips, and sand. The birds were fed twice a day with a diet of meat, grain products, fruits, and vegetables and had access to water *ad libitum*.

### Experimental Procedure

**Figure 1:**
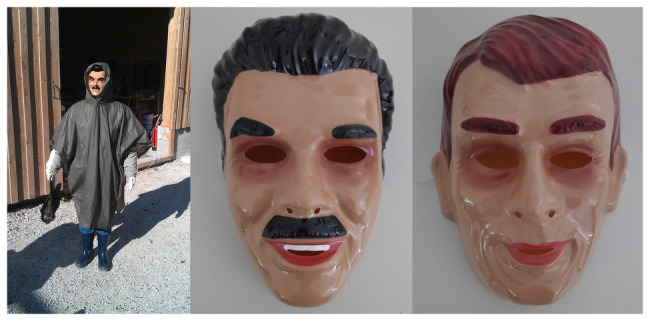
Mask presenter in standardized clothing holding a dead raven. Clothing consists of black rubber boots, white rubber gloves, and an olive plastic poncho. On the right are the black-haired dangerous mask and the red-haired neutral mask.

**Figure 2:**
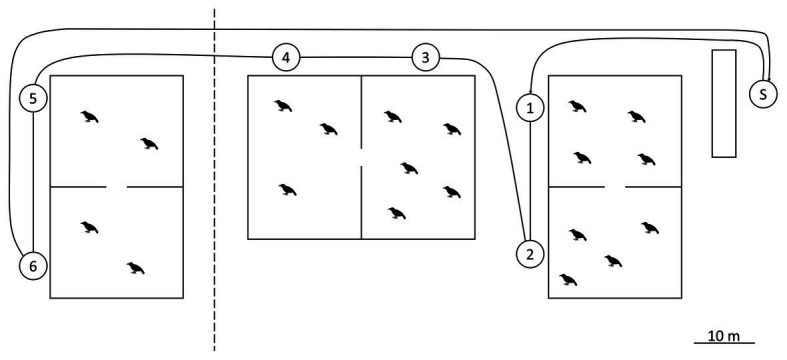
Plan of the aviaries. A barn on the far right provides visual cover for the start of the presentation, marked with “S.” Numbered circles show the presentation locations per aviary. The leftmost aviary was only used after the training phase, when groups got split into pairs. Presentations were only carried out in front of occupied compartments.

The experiment lasted from October 2011 to October 2015 and consisted of three phases. In the initial control phase (October 2011), human presenters wore standardized clothing (gray poncho, rubber boots, and gloves) and one of two masks (Figure 1). The hood of the poncho was worn over the back of the head and the top of the mask to keep the natural hair of the presenters out of view. Wearing one mask, the presenter approached the first aviary and remained still for 2 min. They then moved to the opposite end of the aviary and stood still for another 2 min to ensure that all individuals would have an opportunity to see the mask (Figure 2). The presenter then continued to the second aviary and repeated the procedure. The total duration of the presentation was approximately 10 min. After a break of 30 min, the procedure was repeated with the other mask. Data collection started with a 10-min baseline before each presentation, to ensure no additional events would occur that elicit an alarm response (e.g., birds of prey above the aviary). In such cases, the presentations were postponed. Trials consisted of two presentations per day (one per mask) in the early afternoon and occurred twice a week. Masks were always worn by an actual human, dressed as described above, and the ravens never saw a separate mask alone. Please note that both aviaries were so close together that as soon as the experiment started, the presenter was in view for all individuals. This is also why we did not counterbalance the mask types. Due to the spatial arrangements of the aviaries, the presenter spent the first 4 min in front of the first group (but seen also by the second group), and the next 4 min in front of the second group (but seen also by the first group). However, all individuals had the same exposure time, i.e., 2 × 2 min close-up and 2 × 2 min further away.

In the following training phase (October 2011–November 2011), the black-haired (hereafter “dangerous”) mask was presented together with a dead raven. The dead raven was collected at our field site in the Alps close to the Konrad Lorenz Research Station; it was an adult wild bird killed by captive wolves at the Cumberland Wildpark and thus unfamiliar to our captive ravens at Haidlhof Research Station. The dead raven was shaken; its wings spread and then dropped and picked up at each location. This was an opportunity for the ravens to associate a potential outcome of predation with the “dangerous” mask. There were four trials where a dead conspecific was presented with the dangerous mask. In contrast, the presentation of the neutral mask was performed empty-handed, i.e., neither a dead raven nor any other object was carried by the person when dressed up with this mask. Between every training trial, there was one additional trial where both masks were presented without the dead raven to test for learning speed. Two trials occurred per week.

In the final test phase (November 2011–October 2015), the precision and persistence of these associations were tested by further presentations of both masks without the dead raven. Trials occurred twice per month until May 2012, once a month until November 2013, three times in 2014, and once in 2015.

Across the entire data collection period, both presentations per trial were carried out on the same day and by the same person. We used 17 different presenters for a total of 39 trials. We documented individual scolding durations using video recordings (Canon Legria HF S10, Canon Legria HF S30). Video analysis was performed on PC with the use of Solomon Coder (Péter, 2011).

### Statistics

Analysis was conducted in R (version 3.6.1; R Core Team, 2019) using general linear mixed models (GLMMs) with a beta distribution (using the function “glmmTMB” in the package “*glmmTMB*”; Brooks et al., 2017) and logit link. Theoretically identifiable random slopes and dispersion parameters were assessed using functions provided by Roger Mundry. Variance inflation factors (VIFs) were determined using the function “vif” of the package “*car*” (version 3.0.8; Weisberg and Fox, 2011).

#### Model 1: Learning

During our data collection, the size and number of our groups changed and some additional compartments were included while others were empty and skipped. This resulted in different durations where the mask was in view of the subjects (mean = 223.0 s, *SD* = 88.3 s). We therefore calculated the alarm calling response as proportion of the presentation duration. We linearly scaled our response to a range between 0 and 1 and used a beta distribution. This allowed us to include differences in response intensity which would have been lost in a binomial model.

Prior to analysis, we *z*-transformed all covariates to a mean of 0 and a standard deviation of 1 to increase interpretability and facilitate convergence (Schielzeth, 2010). To provide comparability with other datasets, we list the means and standard deviations for time since training (in days, mean = 356.4, *SD* = 341.3) and group size (mean = 7.3, *SD* = 2.7). We calculated sex ratios for all groups ranging from 0 (all female) to 1 (all male). Finally, we centered and dummy coded all factors with the reference levels being neutral for mask, first for order, female for sex, one for sib-group, and hand-raised for raising. Sib-group only indicates family relation, not necessarily that the siblings were housed or raised together (Table 1).

As response, we used proportion of time spent alarm calling (as described above). As test predictors, we included mask type (dangerous or neutral), sex (male or female), raising type (hand- or parent-raised), and kinship of subject (families indicated by numbers, individuals without siblings by names), and size and sex ratio of the group as fixed effects. As control predictors, we included further fixed effects for order of presentation (first or second presentation of the day), age of the subjects, and days since the last training presentation. As random intercept effects, we included individual and presenter. To reduce type 1 errors, we included theoretically identifiable random slopes (Schielzeth and Forstmeier, 2009; Barr et al., 2013), specifically of age, time since training, mask type, order of presentation, group size and sex ratio within individual and of age, mask type, order of presentation, raising, sex, group size, sex ratio, and sibling-group within presenter. Sample size was 722 observations of 16 individuals. This maximal model did not converge, so we used a reduced model by dropping random slopes of sibling-groups from presenter.

We used the function overdisp.test (provided by Roger Mundry) which returned a dispersion parameter of 0.72 and therefore smaller than 1, confirming that the model is not overdispersed. Slight underdispersion potentially leads to conservative test results and is not generally considered problematic. Collinearity of test predictors was determined for a standard linear model lacking the random effects and appeared to be no issue (maximum VIF: 3.1; Quinn and Keough, 2002).

We conducted a full-null model comparison (Forstmeier and Schielzeth, 2011) to check the overall effect of our test predictors and to avoid cryptic multiple testing. The null model lacked the test predictors but was otherwise identical to the full model (including the same fixed effects for control predictors as well as the same random intercept effects and random slopes). The comparison was based on a likelihood ratio test (function “ANOVA” with “test” argument set to “Chisq”; Dobson, 2002). To investigate differences between sibling-groups, we ran a *post hoc* test by changing the reference levels of “sib-group” (with the command “relevel”) and running separate models for every respective level of sib-group.

#### Model 2: Dominance

To investigate potential influences of dominance on alarm calling behavior, we used a second model including calculated Elo ratings based on won vs. lost conflicts (Albers and de Vries, 2001). This method assigns a new individual rating after every conflict, based on the outcome and the participants’ previous rating. A win against a high-ranking individual is therefore worth more points vs. a low-ranking individual, as is a won high-intensity conflict (e.g., fight) vs. a won low-intensity conflict (e.g., threat). We used data gathered from ongoing, station-wide social focal protocols (5-min individual focal sampling; Altmann, 1974, three times per week) and conducted the analysis in R (using the function “elo.seq” in the package “EloRatings”; Neumann and Kulik, 2020). We set a manual k-factor (i.e., point value) for specific conflict behaviors (fight = 200, chase = 100, challenge = 60, displacement = 40, and threat = 20) and calculated Elo ratings for each individual per group composition which were then scaled to a range of 0–1. Pairs were excluded and video protocols were unavailable for some group compositions and years, resulting in available data for 5 out of 16 group compositions and covering the first 3 years of data collection, reducing our sample size from 722 to 338 observations.

The model formula is similar to model 1, with the addition of a fixed effect for Elo ratings as the only test predictor. As random intercept effects, we again included individual and presenter. We included random slopes of Elo ratings in both individual and presenter but could no longer identify them for age and group size in presenter, so we removed them (this is explained by the reduced sample size covering a smaller number of presentations). Both the dispersion parameter (0.68) and the maximal VIF (3.7) were within acceptable limits. We conducted a full-null model comparison following the same procedure as for model 1 with the null model lacking a fixed effect for Elo ratings, but being otherwise identical to the full model.

#### Model 3: Persistence

Both previous models investigate effects on the overall scolding participation per predictor. To test if the distinction between the masks changed over time, i.e., persistence, we ran a third model using as response the proportion of scolding the bad mask minus proportion of scolding the neutral mask. We again linearly scaled the response between 0 and 1 and fitted a third beta model using the same approach as described above. As test predictors, we included time since training, sex, raising and kinship of the subject, and size and sex ratio of the group as fixed effects. As random intercept effects, we included individual and presenter with random slopes of time since training in individual, raising and sex in presenter, and group size and sex ratio in both. Sibling-group was originally included in presenter but was dropped due to convergence issues. Sample size spanned 361 observations.

There were no issues with overdispersion (dispersion parameter 0.80) or collinearity (maximum VIF 3.1). The null model used for model comparison included only the random intercept effects with the random slopes, but no fixed effects.

## Results

**Figure 3:**
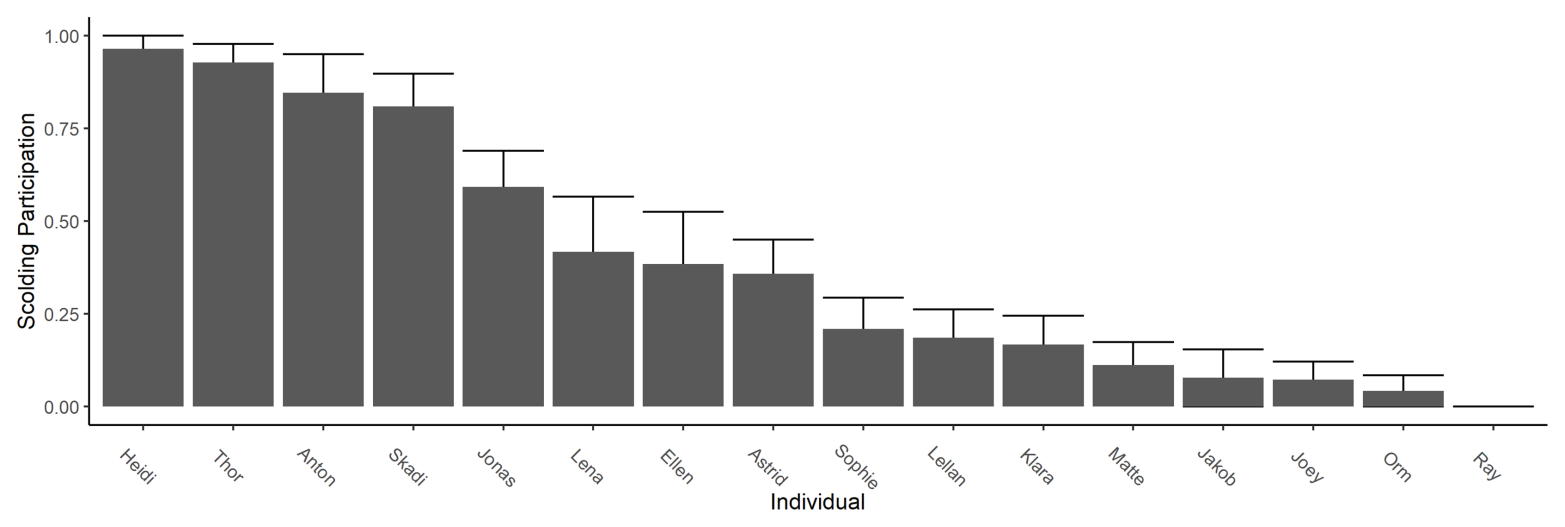
Scolding participation in test phase. Bars show participation in scolding as the proportion of presentations in which the individual produced at least one alarm call. Whiskers show SEs. Across the entire test phase, one individual never participated (male Ray).

**Figure 4:**
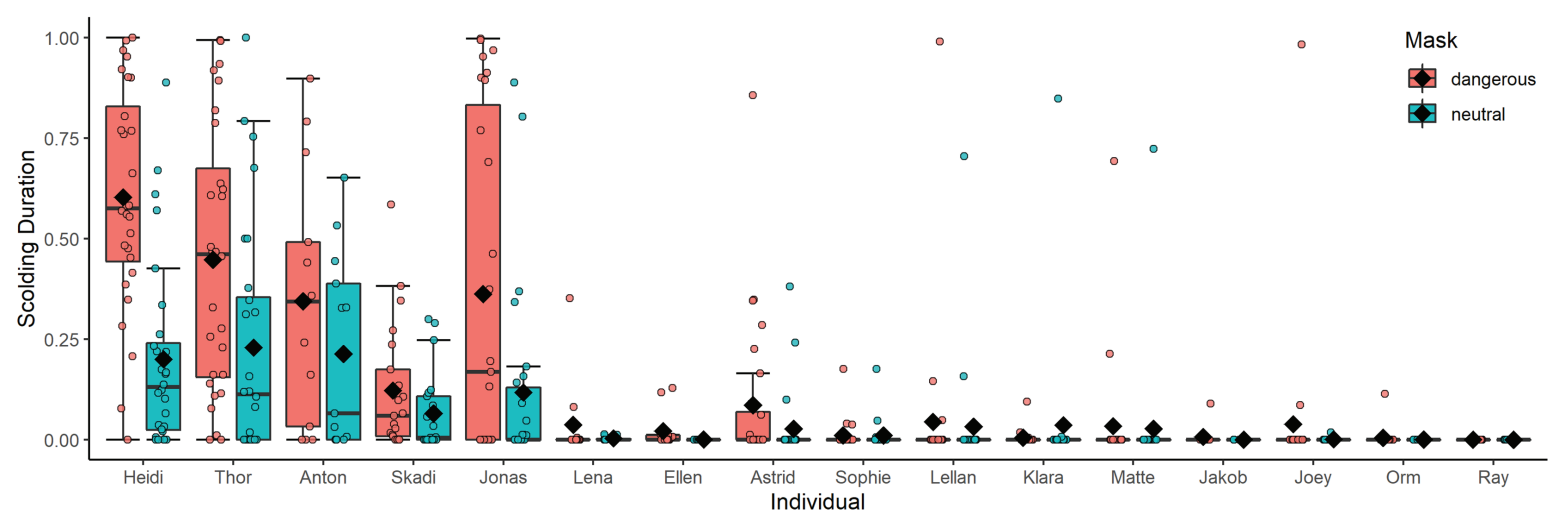
Scolding intensity in the test phase per mask. Individuals are ordered by participation. Black diamonds show means. Only one individual (female Klara) scolded the neutral mask more than the dangerous mask.

All but one raven (male Ray) participated in active scolding of a human wearing a mask in the test phase, even though neither mask was paired with a dead raven at that time any longer. However, individuals varied strongly in their overall scolding participation (whether or not they engaged in scolding; Figure 3) and in their scolding intensity per mask (how long they engaged in scolding; Figure 4). In each of the two original groups, a particular sibling pair (Anton and Heidi in Group A; Thor and Skadi in Group B) took the lead in scolding in respect to both participation and intensity; the males of these pairs were the dominant males in their groups. After the removal of the dominant male of Group A (Anton) and the fusion of the two groups, Jonas became the dominant male and also increased his scolding participation and intensity.

**Figure 5:**
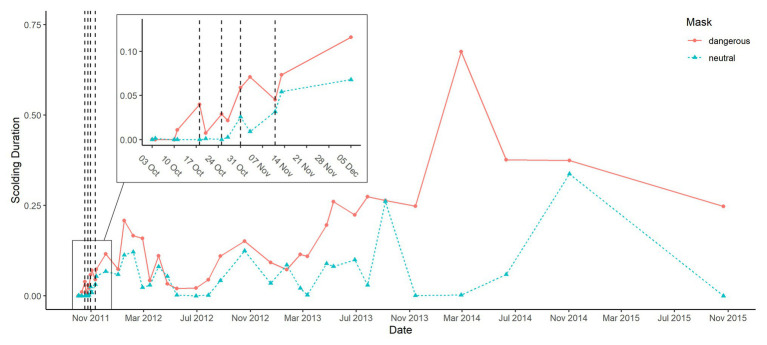
Group averages of scolding response per mask across all phases. The control phase consisted of four trials over 2 weeks, the training phase of four trials with and three trials without dead raven in alternating order over 4 weeks (trials where the dangerous mask was presented while carrying a dead raven are marked with vertical lines) and the test phase consisted of 28 trials over 4 years.

**Figure 6:**
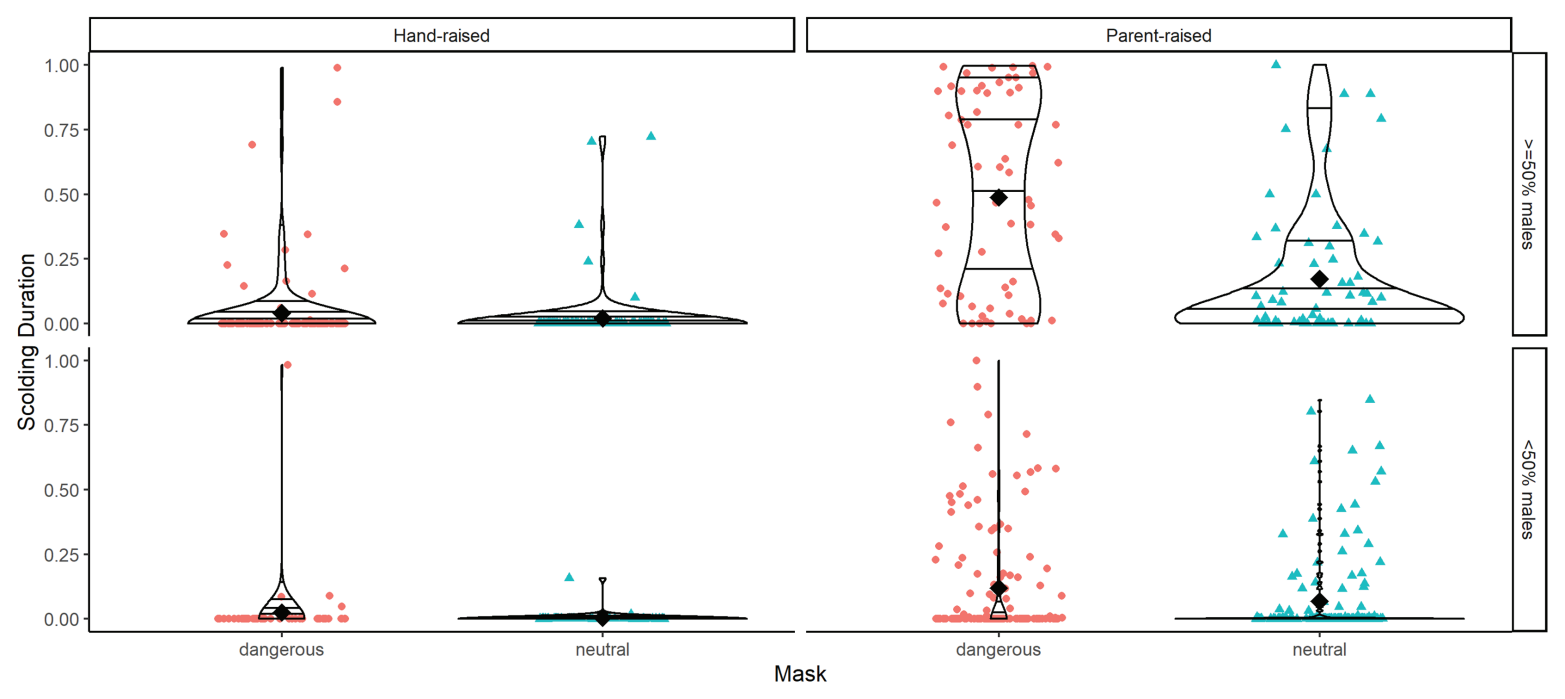
Violin plots of scolding duration as proportion per mask type (dangerous vs. neutral), raising type (hand-raised vs. parent-raised), and group sex ratio (more females than males vs. equal or more males than females). Horizontal lines within the violin plots show quantiles set at 0.25, 0.5, 0.75, and 0.95. Black diamonds show means.

When plotting group averages of scolding response per mask type across time, visual inspection of the graph indicates learning and memory effects (Figures 5, 6). We tested for these effects in addition to effects of individual and social factors (like sex, raising style, kinship, group size, and sex ratio) *via* three statistical models.

### Model 1: Learning

**Figure 7:**
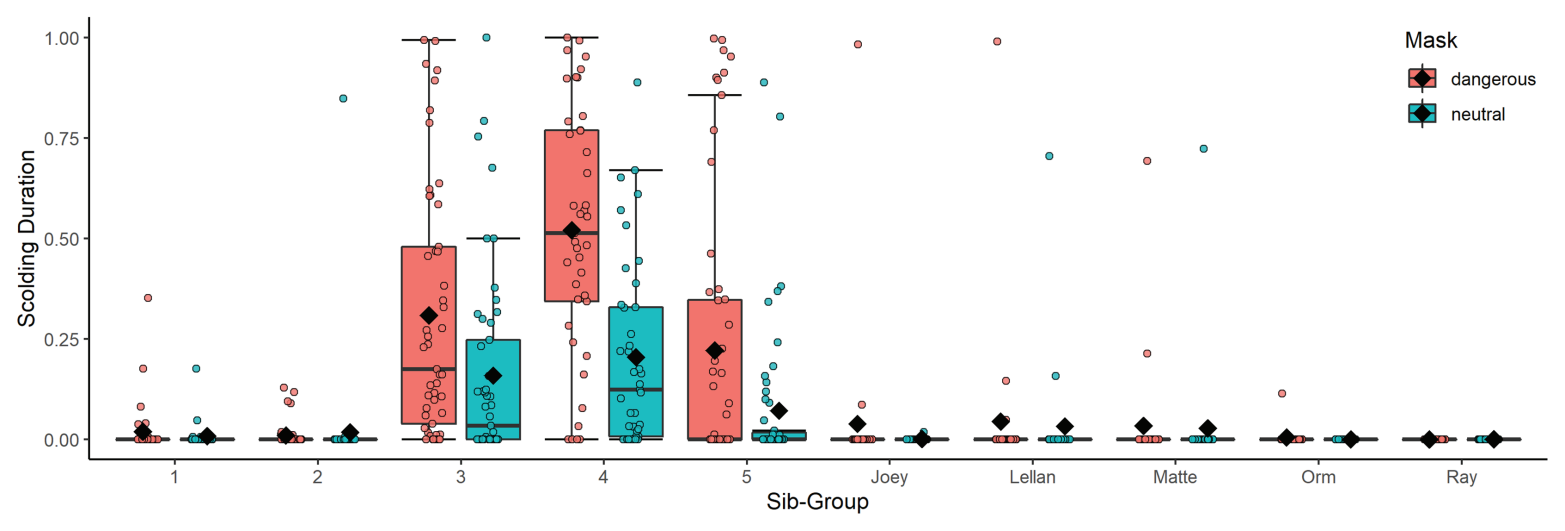
Boxplots of scolding duration as proportion per mask and sib-group. Black diamonds show means. Sib groups indicate only kinship and individuals of the same family were not necessarily housed in the same aviary-compartment or raised together.

Overall, our test predictors (mask type, sex, raising and kinship of subject and size and sex ratio of the group) had a significant impact on scolding response (full-null model comparison: *χ* ^2^ = 49.506, *df* = 14, *p* < 0.001). As expected, subjects spent more time producing alarm calls toward the dangerous mask than toward the neutral mask in the test phase (Table 2). Additionally, social context contributed to individual variation: larger group-sizes correlated with shorter times spent scolding per individual and higher ratios of males in the group with increased scolding duration (Figure 6). Furthermore, individuals that were raised by ravens showed longer alarm responses than those raised by humans (Figure 6). We found no significant effects for sex. Finally, there were differences in scolding duration between sibling-groups (Figure 7). *Post hoc* testing revealed significant differences for group 4 when compared to groups 1, 2, 5 and Joey (*p* < 0.001 in all cases) and a trend for the comparison of groups 5 and 2 (*p* = 0.054).

**Table 2:** Output from Model 1 on long-term memory.

Fixed effects	Estimate	*SE*	*z* value	*p* value	
(Intercept)	−4.67	0.98	−4.76	<0.001	***
Dangerous mask	0.43	0.18	2.40	0.017	*
Order 2^nd^	−0.26	0.17	−1.53	0.126	
Sex male	−0.08	0.16	−0.48	0.631	
Age	1.82	1.73	1.05	0.293	
Sib-group 2	0.41	0.27	1.54	0.123	
Sib-group 3	1.72	0.21	8.17	<0.001	***
Sib-group 4	−0.04	0.18	−0.20	0.843	
Sib-group 5	2.86	1.93	1.48	0.139	
Sib-group Joey	0.38	0.35	1.08	0.282	
Sib-group Lellan	2.50	1.93	1.29	0.197	
Sib-group Matte	2.54	1.95	1.30	0.193	
Sib-group Orm	2.53	1.95	1.30	0.195	
Sib-group ray	2.47	1.95	1.27	0.205	
Raising parent	0.78	0.29	2.68	0.007	**
Sex ratio	3.24	0.73	4.44	<0.001	***
Group-size	−0.11	0.04	−2.66	0.008	**
Time since training	−1.66	1.48	−1.13	0.260	

General linear mixed model (GLMM) output showing fixed effects with response as proportion of scolding. Age and time since training were z-transformed, the rest dummy coded with the reference categories being neutral (for mask), first presentation (for order), 1 (for sib-group), and hand-raised (for raising). Higher sex ratios indicate more males. *N* = 722. Significance codes: <0.1; * < 0.05; ** < 0.01; *** < 0.001.

### Model 2: Dominance

**Figure 8:**
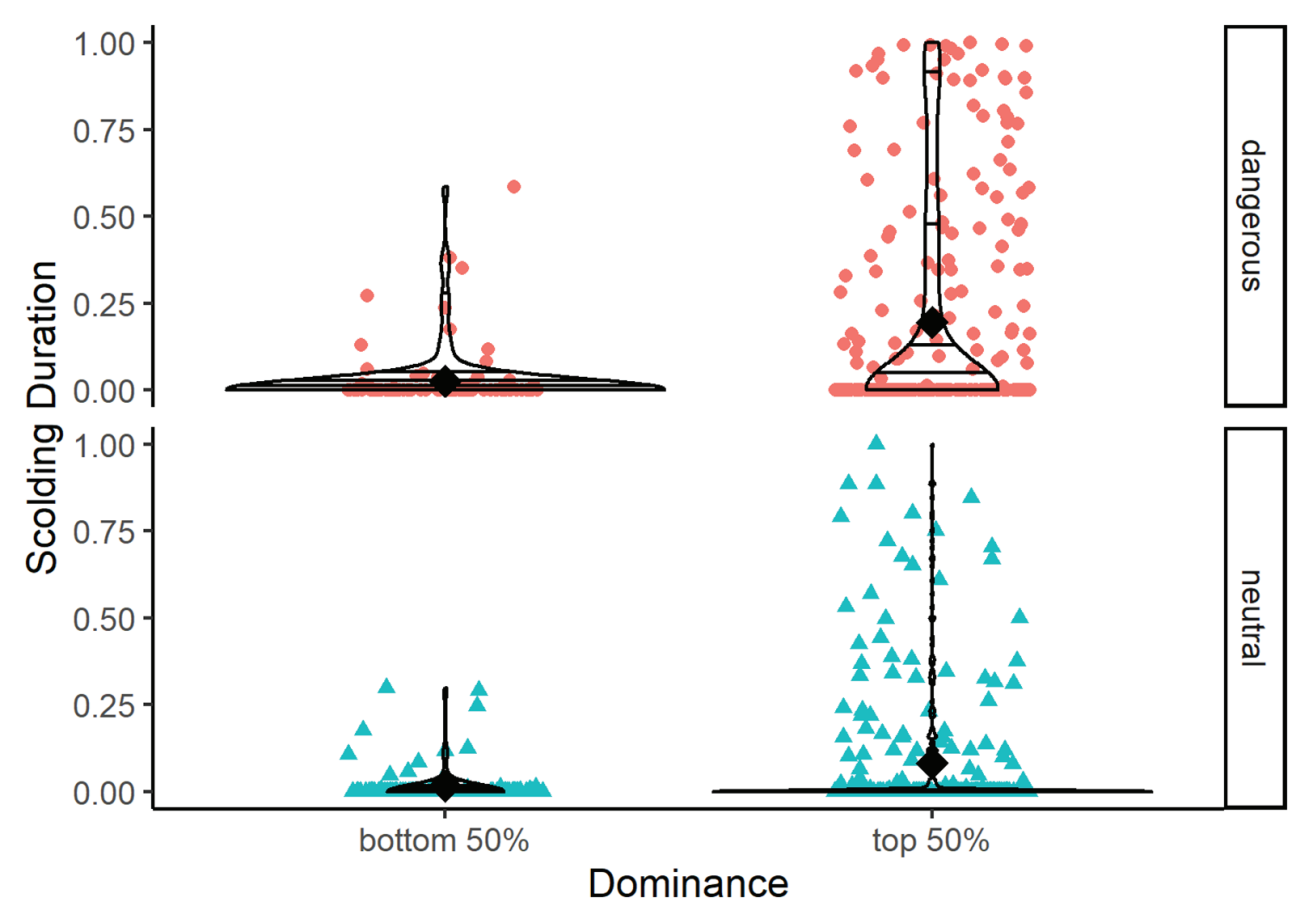
Violin plots showing scolding duration as proportion per mask type (dangerous vs. neutral) and dominance (top 50% of dominant individuals vs. bottom 50%). Horizontal lines within the violin plots show quantiles set at 0.25, 0.5, 0.75, and 0.95. Black diamonds show means.

The full-null model comparison, with Elo ratings being the only test-predictor, was significant (*χ* ^2^ = 8.398, *df* = 3, *p* = 0.038). Focusing on the 3-year time period for which information on dominance relationships was available, we found that individuals with higher Elo ratings showed longer scolding durations (Table 3, Figure 8).

**Table 3:** Output from Model 2 on dominance.

Fixed effects	Estimate	*SE*	*z* value	*p* value	
(Intercept)	−9.74	6.57	−1.48	0.138	
Mask dangerous	0.28	0.12	2.29	0.022	*
Order 2	−0.15	0.10	−1.42	0.156	
Sex male	−1.56	0.41	−3.85	<0.001	***
Age	−3.04	1.80	−1.68	0.092	
Sib-group 2	0.16	0.33	0.47	0.635	
Sib-group 3	1.56	0.29	5.37	<0.001	***
Sib-group 4	−0.26	0.22	−1.18	0.240	
Sib-group 5	−3.86	3.41	−1.13	0.257	
Sib-group Joey	0.95	0.51	1.87	0.061	
Sib-group Lellan	−4.49	3.33	−1.35	0.178	
Sib-group Matte	−3.57	3.37	−1.06	0.289	
Sib-group Orm	−4.07	3.36	−1.21	0.226	
Sib-group ray	−3.78	3.36	−1.12	0.261	
Parent-raised	1.42	0.99	1.44	0.150	
Sex ratio	−1.63	7.17	−0.23	0.820	
Group-size	0.92	0.50	1.86	0.063	
Time since training	0.98	0.91	1.08	0.279	
Dominance	2.41	0.51	4.75	<0.001	***

GLMM output showing fixed effects with response as proportion of scolding. Elo ratings for dominance were scaled from 0 to 1, age and time since training were z-transformed, the rest dummy coded with the reference categories being neutral (for mask), first presentation (for order), 1 (for sib-group), and hand-raised (for raising). Higher sex ratios indicate more males. *N* = 338. Significance codes: < 0.1; * < 0.05; ** < 0.01; *** < 0.001.

### Model 3: Persistence

Other than models 1 and 2, we now used as response the difference in scolding duration between the masks (dangerous minus neutral), rather than scolding duration in general. The combination of test predictors (time since training, sex, raising and kinship of the subject and size and sex ratio of the group) had a significant effect on mask-distinction (full-null model comparison: *χ* ^2^ = 29.096, *df* = 14, *p* = 0.010). Notably, we found no changes in distinction between the masks across time in the test period (Table 4, Figure 5). Parent-raised individuals responded to the dangerous mask stronger than hand-raised individuals (Figure 6). The model also revealed that some sibling-groups discriminated better than others (Figure 7), and overall the discrimination was better when more males were present (Figure 6). We found no significant effects of caller sex or group size.

**Table 4:** Output from Model 3 on memory.

Fixed effects	Estimate	SE	*z* value	*p* value	
(Intercept)	−0.85	0.37	−2.29	0.022	*
Sex male	0.06	0.12	0.51	0.609	
Sib-group 2	0.40	0.15	2.59	0.010	**
Sib-group 3	0.63	0.12	5.43	<0.001	***
Sib-group 4	−0.07	0.11	−0.64	0.522	
Sib-group 5	0.08	0.12	0.65	0.515	
Sib-group Joey	0.50	0.19	2.61	0.009	**
Sib-group Lellan	0.46	0.19	2.39	0.017	*
Sib-group Matte	0.36	0.25	1.42	0.156	
Sib-group Orm	0.40	0.26	1.57	0.115	
Sib-group ray	0.39	0.26	1.53	0.125	
Raising parent	0.57	0.16	3.52	<0.001	***
Sex ratio	1.35	0.44	3.04	0.002	**
Group-size	−0.03	0.03	−0.87	0.387	
Time since training	0.09	0.07	1.29	0.197	

GLMM output showing fixed effects with response as difference in proportion of scolding per mask (dangerous – neutral). Age and time since training were z-transformed, the rest dummy coded with the reference categories being neutral (for mask), first presentation (for order), 1 (for sib-group), and hand-raised (for raising). Higher sex ratios indicate more males. *N* = 361. Significance codes: < 0.1; * < 0.05; ** < 0.01; *** < 0.001.

## Discussion

Captive ravens quickly learned to distinguish human experimenters wearing one of two masks, whereby the “dangerous” mask was initially paired with the presentation of a dead conspecific and the neutral mask was not. In subsequent tests without a dead raven, ravens scolded more toward humans wearing the dangerous mask than the neutral mask; furthermore, they continued to do so over a 4-year period without further experimental reinforcement. Despite having received the same amount and quality of exposure, individual birds differed strongly in how often and/or how long they participated in scolding the masked humans. This inter-individual variation was largely explained by social factors and fairly consistent across experimental presentations in socially stable situations. Later changes in the individuals’ scolding participation and/or intensity coincided with changes in group composition and pair formation.

### Learning

Ravens quickly learned to distinguish between humans based on their facial features, which is in line with the results of previous studies on other corvids (Levey et al., 2009; Marzluff et al., 2010; Lee et al., 2011; Davidson et al., 2015). As in American crows (Swift and Marzluff, 2015), seeing a dead conspecific being carried by a human was enough to form an association between this putative predation event and the facial features of that person, i.e., his or her mask. Note that we used different human presenters across the experiment, but always had the same person present both masks during each test round; this procedure makes it unlikely that the ravens based their discrimination on any other human features (body shape, movement, odor, etc.) but the masks. During our control phase before training, we observed hardly any scolding response to either mask. Thus, we can rule out that the ravens had a general aversion to masked humans or an initial preference or dislike for one mask over the other. Hence, we argue that the ravens assigned different threat levels to the two masks as a result of the four training trials with a dead conspecific. However, as our neutral mask was always presented empty-handed, the possibility remains that the ravens’ assignment of different threat levels might be generally caused by a human carrying an item (and not a dead raven).

### Dominance and Social Features

A noteworthy result of our study was the high individual variation in scolding participation, despite the equal and highly controlled exposure experienced by all birds. This variation could be explained by a mix of factors: Model 1 revealed effects of kinship, i.e., sibling groups participating either strongly or weakly in scolding (Figure 7). It remains unclear what the basis for these sibling effects might be, e.g., genetics, epigenetics, or social constraints (Champagne, 2008; Oliveira, 2009; Taborsky et al., 2012). We also found a negative effect of group size, indicating a potential dilution effect (Hamilton, 1971; Foster and Treherne, 1981; Lima and Dill, 1990).

Unlike Buitron (1983) we found no effects of caller sex, but we saw higher scolding durations in groups with higher ratios of males, possibly indicating male competition using scolding as status signal. This interpretation is further supported by dominant individuals producing more alarm calls (status signals), and the fact that in ravens males are typically more dominant than females (Harriman and Berger, 1990; Stöwe et al., 2006). Model 2 showed dominance to be one of the strongest predictors for scolding intensity overall. Previous studies on captive jungle fowl (*Gallus gallus*) confirmed higher mating chances for males that produce more alarm calls (Wilson et al., 2008), and showed positive correlations of anti-predator behavior and dominance (Pizzari, 2003). Studies on another corvid, the Siberian Jay, showed increased mobbing intensity for breeding alpha males within kin-groups, specifically in presence of their own offspring (Griesser and Ekman, 2005). A follow-up study argues that mobbing events of adult conspecifics would provide opportunity for predator-learning to the offspring (Griesser and Suzuki, 2017). We found a similar increase in scolding in paired adults, but in the absence of any offspring. We thus interpret the intensive scolding by dominant ravens to have other functions than predator-learning in offspring, like signaling status and/or quality (Slagsvold, 1984; Ellis, 2009; Tanager, 2011). The latter is supported by a study showing that males across 19 species increase their mobbing intensity in the presence of females (da Cunha et al., 2017a), and a comparison of 145 species of birds concluding that different social systems do not influence mobbing behavior (da Cunha et al., 2017b).

We also observed high-ranking individuals to aggressively challenge low-ranking individuals for producing intense scolding bouts (personal observation). However, because individuals tended to be close to the presenter while scolding, an alternative explanation would simply be redirected aggression toward the nearest subordinate group member (instances of re-direction have been observed in captive and free-ranging ravens, but not systematically studied). These dilution or suppressor effects could be responsible for the low scolding responses and failure to distinguish between the masks in some individuals, rather than a failure in learning to identify the masked human as potential threat. Disentangling these effects is not possible in our paradigm, but would be an interesting line of investigation for a follow-up study testing participating individuals in separation. If individuals distinguish between the masks in isolation, it would rule out a failure to learn, and support the presence of dilution or suppressor effects while in the group. By testing focus individuals in dyads with higher vs. lower ranking individuals, one could investigate dominance effects in more detail.

Finally, Model 1 also revealed an effect of rearing style, with parent-raised birds scolding the human presenters more readily and intensively. This is in accordance with the substantial literature on early life experiences, often showing long-term effects (Hemetsberger et al., 2010; Boucherie et al., 2020). The upbringing by human foster parents likely made them less receptive for treating humans as potential predators. However, when hand-raised ravens engaged in scolding, they performed similarly to parent-raised birds and discriminated accurately between masks.

### Patterns Across Time

Scolding intensity (to either of the masks) was rather low during training, and at the beginning of testing, but increased throughout the testing phase (Figure 5). A similar pattern has been observed in other avian species like mockingbirds (Levey et al., 2009), crows (Marzluff et al., 2010), magpies (Lee et al., 2011), and jackdaws (Davidson et al., 2015). One way to explain this pattern is that the presenters’ disappearance after being scolded acts as reinforcement for future scolding (Knight and Temple, 1986; Griffin, 2004; Marzluff et al., 2010). The increased number of visits by masked persons could also elevate the perceived threat level, as reported for magpies (Redondo and Carranza, 1989; Lee et al., 2011). Conversely, one might argue that the repeated appearance of the dangerous person without any consequences reduces the perceived threat level, resulting in less fearful birds being more liberal in their scolding response (Marzluff et al., 2010). It is not possible to test these hypotheses with our current dataset, but additional presentations of the dangerous mask while carrying a dead conspecific, could again elevate a potentially lowered threat level. If afterward the scolding intensity did not decrease again, we could rule out that the birds were no longer perceiving the dangerous mask as a serious threat.

The discrimination between masks was hardly affected by the time elapsed since training in the experiment, suggesting that (at least some) ravens remembered the putative predation events for 4 years. While the dangerous mask elicited longer scolding durations throughout the study, we did notice some generalization, and thus increased calling, toward the neutral mask toward the end of the study period. This has also been observed in related studies on other corvids (Marzluff et al., 2010; Davidson et al., 2015), and in our case could be explained by the similarities between the two test conditions like identical clothing of the human presenters and the shared traits of the masks (e.g., their stiffness and glossiness). Given the low costs of scolding a masked person, and potentially high rewards of avoiding future predations (Marzluff et al., 2010), it is quite noteworthy that the ravens’ responses to the neutral mask remained distinguishable from those to the dangerous mask for the entire study period.

While in all social constellations the dominant males of the groups took the lead in scolding, the majority of group members participated at low levels. The dominant males were accompanied in scolding by their siblings before they reached maturity (first 1–2 years of the study) and, after pair formation, by their female partners. Pair formation seemed to boost participation in scolding of (previously) subordinate females and males alike, which fits the finding that pair formation accompanies a rise in dominance status (Braun and Bugnyar, 2012). Taken together, these individual-level patterns support the notion that the social context is central to understanding ravens’ participation in anti-predator behavior. While ravens seem to be fairly plastic in how much they contribute to scolding, their degree of consistency over time seems to be tied to social opportunities and constraints (see Lima and Dill, 1990 for a review; in birds: Hogstad, 1988; in mammals: Atwood and Gese, 2008; in fish: Brown et al., 2009).

### Concluding Summary

Literature on heterospecific individual recognition is relatively rare, with the exception of recognition of human faces, which has been shown in variety of species, ranging from mammals, birds, and reptiles to invertebrates like octopuses and honeybees (Taylor and Davis, 1998; Davis, 2002; Dyer et al., 2005). However, testing methodology varied in most of these studies, which led to difficulty in comparing their results and conclusions (Dittrich et al., 2010). With the current study, we add to the recent literature investigating predator learning by using (masked) humans, reflecting a relatively standardized method of testing (Levey et al., 2009; Marzluff et al., 2010; Lee et al., 2011; Davidson et al., 2015). Similar to previous findings, we observed rapid learning after only four training presentations, resulting in behavior that reliably distinguished between the masks over 4 years. Because we worked with captive individuals, we obtained valuable additional information concerning large individual variation in scolding participation, intensity, and to some extent, level of discrimination between masks. This variation is mainly explained by social factors, notably dominance, and relative number of males in the group.

Although ravens regularly exploit human resources (Webb et al., 2004; Loretto et al., 2016), they typically do not live in densely human populated urban areas. The latter has been discussed as a key variable in explaining the ability of animals to discriminate between humans on an individual basis (e.g., Davis, 2002). We may thus wonder why ravens could (easily) come to recognize individual humans in the current study? On one hand, not only the frequency of exposure to humans may matter, but also the variation in human behavior toward the species in question. As scavenger, ravens have been exposed to humans as both “food providers” (that deliver garbage, animal kills etc.) and “predators” (that shoo them away or even hunt them) within their individual lives and for many generations (hundreds or thousands of years, Marzluff and Angell, 2005). They may thus have developed a predisposition to pay attention to individual features of humans that go together with their behavior. Selection for paying attention to human facial features has also been shown in domestic animals like dogs (Huber et al., 2013). On the other hand, discriminating between heterospecifics may come as a by-product of conspecific recognition, which has been proposed to be adaptive in social species (Tibbetts and Dale, 2007). Ravens may simply extend this ability to heterospecific individuals, which come to interact with them in relevant ways, i.e., as providers or predators. The latter interpretation would fit to several other species, for which differentiation among human individuals has been reported (Davis, 2002). Our study implies that social context shapes the expression of birds’ knowledge about humans (or potential predators in general). Further investigation of the factors explaining the consistency and plasticity of inter-individual variation in corvids’ behavior toward humans provides a promising line of future research.

## Data Availability Statement

All datasets presented in this study are included in the article/Supplementary Material.

## Ethics Statement

The animal study was reviewed and approved by Animal Ethics and Experimentation Board Faculty of Life Sciences University of Vienna.

## Author Contributions

TB and CB designed the study. CB collected and analyzed the data and drafted the manuscript under the supervision of the other authors. TB and WF provided critical revisions to the manuscript. All authors approved the final version of the manuscript for submission.

### Conflict of Interest

The authors declare that the research was conducted in the absence of any commercial or financial relationships that could be construed as a potential conflict of interest.
